# A proof of principle investigation of a novel enzyme formulation on dental calculus deposition: a 4-week randomized human clinical trial

**DOI:** 10.1186/s12903-024-04498-x

**Published:** 2024-06-22

**Authors:** Kimberly R. Milleman, Greg Deener, Jeffery L. Milleman, Barbara Mish, Reinhard Schuller, Dennis Mynarcik

**Affiliations:** 1 Salus Research, Inc., 1220-4 Medical Park Dr., Fort Wayne, IN 46825 USA; 2 Pontis Biologics, Inc., Long Island High Technology Incubator, 25 Health Sciences Dr., Stony Brook, NY 11790 USA; 3 Reinhard Schuller Consulting, 37 Poplar Heights Dr., Toronto, ON Canada

**Keywords:** Calculus, Enzymes, Brushing, Volpe-Manhold Index (V-MI), Adverse, Safety, Formulation

## Abstract

**Background:**

The dissolution of dental calculus, safely and at home, is among the more challenging issues facing the over-the-counter healthcare industry. Pontis Biologics, Inc. has developed novel model of calculus development and structure and has formulated a dentifrice (Tartarase™) using digestive enzymes as active ingredients that is shown to dissolve dental calculus in this Proof of Principle clinical trial.

**Methods:**

This investigation was designed to evaluate the safety and efficacy of a novel enzyme formulation to remove existing calculus deposits in 4 weeks, measured using the Volpe-Manhold Index (V-MI) on lingual surfaces of 6 lower anterior teeth. The test formulation was compared to Crest Cavity Protection, as a control dentifrice. A total of 40 randomized test subjects began the study with 20 assigned to the control dentifrice and 20 assigned to the Tartarase groups (ten each, one brushing with Tartarase twice daily and one brushed with Tartarase and wore a dental tray filled with Tartarase for 30 min then brushed again with Tartarase, once daily).

**Results:**

The Crest group experienced a 12% increase in calculus, in contrast to the results of both Tartarase groups that experienced a 40% reduction in calculus in 4 weeks of unsupervised at home use of the Tartarase toothpaste formulation.

**Conclusions:**

This proof of principle study demonstrates that a dentifrice, formulated along the lines of the Tartarase material, is capable of combating calculus accumulation using the same oral hygiene habits that are common worldwide.

**Trial registration:**

This trial was registered retrospectively at clinicaltrials.gov and has the Unique Identification Number: NCT06139835, 14/11/2023.

## Background

The World Health Organization estimates oral diseases affect nearly 3.5 billion people globally with untreated dental caries being the most common health condition, and 1 billion people having severe periodontal diseases. The estimated number of cases of oral diseases globally is about 1 billion higher than mental disorders, cardiovascular disease, diabetes mellitus, chronic respiratory diseases and cancers combined. Moreover, it is the most prevalent health problem in high income nations [1]. In the United States, data from the 2018 NHANES estimated that 42% of dentate US adults have periodontitis [2] and over 25% of adults in the United States have untreated tooth decay [3]. Importantly, oral health diseases exert systemic manifestations beyond the oral cavity such as diabetes, cardiovascular disease, and pre-term babies with low birth weights [4]. The linkage is not precisely defined, but commonly associated with a chronically inflammatory state, originating with periodontal inflammation. Oral biofilms are the main etiologic factor for a variety of oral diseases such as dental caries, and periodontal diseases. An integral feature of biofilms, including dental plaque, is extracellular DNA (eDNA) that aids in the formation of the gel nature of plaque [5] and serves as a nidus for the process of mineralization into calculus. DNA is a known chelator, along with the negatively charged salivary mucin glycoproteins, bind the abundant calcium, present in saliva at 1.5 mM, making calculus rugged and difficult to remove at home. Bacteria living in dental biofilms are the primary etiological factors of periodontal diseases [6]. In addition, bacterial species living in dental biofilm digest glycoprotein polysaccharides to harvest and metabolize sugar [7] and produce acids that reduce the biofilm fluid pH. This leads to a mineral discrepancy between acidic biofilm fluid and tooth, resulting in loss of tooth mineral composition, clinically noted as loss of tooth structure and cavitations [8]. The control and prevention of dental biofilms, plaque, and dental calculus accumulations are widely held as conditions that lead to a healthy oral environment. Unilever has performed a landmark study on the use of an enzyme-containing dentifrice (Zendium) that produces antimicrobial substances in the oral environment. These substances improve the quality of the oral microbiome, but do not impact calculus accumulation [9].

Oral prophylaxis, especially tooth brushing, is a basic and daily custom for almost all people, even in developing countries, and its aim is to remove dental plaque. However, more than 40% of plaque will not be removed, even by a well-trained person [10], and, in general proceeds to become mineralized forming dental calculus. Current toothpastes are ineffective at preventing dental calculus formation [11]. At best they decrease the rate at which calculus forms. For example, a Cochrane Collaboration publication stated after six months of use, triclosan/copolymer toothpaste participants had a mean total calculus of 12.49 mm, and the control group was 14.61 mm. Both groups formed measurable amounts of dental calculus though the triclosan/copolymer toothpaste group had 15% less dental calculus formed (it slows but does not prevent the formation of dental calculus) [12].

Pontis Biologics has constructed a model of dental calculus development that is minimalist in nature, supported by the data presented in this publication, as well as laboratory data generated with pulverized canine calculus (manuscript in preparation). Our model predicts that the enzymatic hydrolysis of eDNA and proteins, the substances that form an integrated matrix supporting the calculus structure, lead to the dismantling of calculus, and to the release of bulky bacteria and associated debris that are entrapped in a calcium-mediated crosslinked complex of negatively charged eDNA and mucin, intertwined with denatured protein, reducing the thickness, and reducing the outer dimensions of the existing calculus. Other, more complex models have been proposed for calculus structure, incorporating macromolecular associations that are supported by molecular analysis [13]. However, the Pontis model focuses on the minimum grouping of molecular constituents that serve to reinforce the structure of calculus. For any non-mechanical method of calculus removal to be efficacious it must, during its application, remove more calculus than can be accumulated before the next treatment. The data we present clearly demonstrates that this has been achieved. The data also suggests that acceptable results will likely be achievable with a reduced quantity of the active ingredients employed in the present formulation. Thus, Pontis Biologics, with their proprietary and patented combination of enzymes (i.e., Tartarase™) has been demonstrated to destabilize and breakdown dental calculus. The purpose of this proof of principle study was to determine the efficacy and safety of the Tartarase formula to decrease calculus formation in an examiner-randomized human clinical trial.

## Methods

This Proof of Principle human clinical investigation was a parallel group, examiner-blind, randomized, three treatment clinical trial to evaluate the safety and efficacy of a novel enzyme formulation (i.e., Tartarase) to decrease existing calculus deposits in 4 weeks, measured using the Volpe-Manhold Index (V-MI) on lingual surfaces of 6 lower anterior teeth [14]. Among the secondary objectives were an evaluation of safety, assessed by examination of the gingival tissues (free and attached), hard and soft palate, oropharynx, buccal mucosa, tongue, floor of the mouth, labial mucosa, mucobuccal/mucolabial folds, lips, and perioral area. In addition, a questionnaire was selected using a five-point subjective scale to evaluate oral cleanliness (1 = least clean and 5 = cleanest) [15] and was included as an exploratory endpoint. Safety assessments and oral cavity examination were conducted at all visits. Forty subjects were consented and enrolled with 20 randomized to the control dentifrice (Crest Cavity Protection) and 20 subjects were assigned to the two Tartarase treatment groups.

The research study was approved by the U.S.IRB (U.S.IRB2022SRI/10) in accordance with ICH Guidelines E6 and the USA Food and Drug Administration and conducted by Salus Research, Inc., (an American Dental Association (ADA) Qualified Independent Research Site). The investigational product did not contain fluoride and the IRB requested all participants brush twice daily with a fluoride containing toothpaste.

Recruitment began May 25, 2022 and the study was concluded July 6, 2022.

The inclusion criteria for study subjects:

- All participants were provided with a written consent to participate in the study, were at least 18 years of age, and they were in good general health, as determined by the investigator/designee based on a review of the health history/update for participation in the study
- All agreed to not participate in other oral/dental product studies during the course of this study, and use only the assigned oral hygiene products during the entire study (including toothbrushes, toothpastes, home remedies, floss, chewing gum, mouthwashes, tongue cleaners, etc.)
- All agreed to refrain from the use of any elective dentistry (including non-study dental prophylaxis) until the study was completed.
- All agreed to refrain from the use of non-study oral hygiene and whitening products.
- All had six mandibular anterior teeth with no crowns or veneers, agreed to comply with the study procedures and schedule, including the follow up visits.
- All had at least a total of 9mm of dental calculus on the lingual surfaces of mandibular anterior six teeth using the Volpe-Manhold methodology [14] and reported that they had received a dental cleaning in previous 2-6 months.

The exclusion criteria for the study subjects:

- They will have a medical condition requiring antibiotic premedication prior to dental procedures.
- They regularly used chlorhexidine mouth rinse.
- They have any oral condition or pathosis that could interfere with study compliance and/or examination procedures (e.g., widespread caries, chronic neglect, advanced periodontal disease.)
- They have current or history of oral cavity cancer or oropharyngeal cancer.
- They are pregnant or nursing by subject report.
- They do not brush their teeth regularly.
- They have any condition that might make it unsafe for the subject to participate in this study, at the discretion of the investigator.

The data were collected at the dental facility of Salus Research, Inc. During the initial visit, participants were assessed to ensure at least 9 mm of dental calculus was present on the lingual surfaces of the six mandibular anterior teeth, assessed by the V-MI [14] and eligible subjects were evaluated for demographics, medical history, and concomitant medications. The 40 subjects who qualified were randomly assigned to one of the three study groups by using a randomization schedule developed and maintained by an independent statistician. Subjects were stratified by baseline lingual V-MI dental calculus scores and gender. Half of the test subjects (*n* = 20) were randomized to the control group and 10 to each of the Tartarase arms (Tartarase Brush only and Tartarase Brush + tray). All subjects performed their first use supervised at the research site then unsupervised during the 4-week test. For each subject, assessment data was collected at baseline, 2 and 4 weeks. All test subjects brushed twice daily with the control toothpaste Crest® Cavity Protection Cool Mint Gel, 0.243% Sodium Fluoride using the same an ADA reference soft manual toothbrush. Test group assignments were as follows:

1. Control (20 subjects): Brush twice daily for two minutes with ADA toothbrush and Crest.
2. Tartarase Brush (10 subjects): Brush twice daily with Tartarase and twice with Crest.
3. Tartarase Tray (10 subjects): A custom dental tray was made for all Tartarase Tray subjects. Test subjects brushed 1 minute with Tartarase then applied Tartarase using the custom tray (worn once daily for 30 minutes). This procedure was followed by another brushing with Tartarase for 1 minute. The Tartarase brush/tray/brush was performed once daily. Participants also brushed twice daily with Crest.

### Tartarase formulation

The formulation was prepared in two batches, each sufficient for 2.0 ml/day for 14 days for 20 participants. Ceteareth-25 (0.23%), glycerol (13.6%), Xanthan gum (0.28%), sorbitol (4.4%) and hydrated silica (11%). DNase 1 (197, 326 Kunitz units/ml), and chymotrypsin (3,333 units/ml). The enzymes were obtained from Worthington Biochemicals. Because this was a 4-week study, the decision was made to formulate the test product using twice the levels per unit volume previously shown to be efficacious in preclinical studies using canine calculus.

### Sample size and randomization

The power to detect plaque treatment differences would be 90% with 20 evaluable subjects receiving active treatment. This sample size would be able to detect a 25% improvement in the Plaque Index for the active treatment compared to control. A simple randomization with a block size of 3 was used to generate the random allocation sequence for the three treatments. The sequence was generated using statistical software SAS 9.4 for Windows. The full analysis data set was used in each analysis and the numbers are given in Table 1. The recorder and examiner were blinded to treatments. The study coordinator was unblinded as she was the one assigned to randomize subjects into treatment groups.

**Table 1:** Baseline Characteristics

	Crest Control	Tartarase Brushing	Tartarase Tray	Total
Randomized	20	10	10	40
Completed	20	8	9	37
Mean V-MI at Baseline	17.3	16.6	17.5	
Mean Age in Years	50.2	58.9	42.3	50.4 (*p* = 0.234)
Gender				
Male	9 (45.0%)	5 (50.0%)	4 (40.0%)	18 (45.0%)
Female	11 (55.0%)	5 (50.0%)	6 (60.0%)	22 (55.0%)
Race/Ethnicity				
White / Caucasian	15 (75.0%)	10 (100.0%)	8 (80.0%)	33 (82.5%)
Black / African Heritage	3 (15.0%)		2 (20.0%)	5 (12.5%)
Multiracial	2 (10.0%)			2 (5.0%)
Mean times brush teeth daily	1.8	1.9	1.6	1.7

This table lists the baseline characteristics, including the mean Volpe-Manhold Index (V-MI) of the different treatment groups, demographic data of the different treatment groups and the normal brushing habits of those in the different treatment groups

### Efficacy assessments and statistical analysis

Efficacy was determined by the change is dental calculus within treatment groups (longitudinal analysis) and between groups as compared to the control group following 4 weeks of treatment. Additionally, efficacy in the Tartarase treatment groups was determined by the prevention of calculus accumulation, assessed by no significant increase in calculus score as compared to baseline. The primary outcome was calculus abundance using the V-MI, and all other variables are considered secondary. The measurement is performed on the lingual surface of the 6 mandibular anterior teeth. The calculus scores for this index were summed to provide a total score per mouth at each clinical examination. The primary timepoint was at the conclusion of the trial after 4 weeks of product use.

Data from all subjects who completed the final assessment were used in the analysis. The data for each scoring index was also analyzed by analysis of covariance using the baseline data as the covariate. The covariate (baseline data) was included in the statistics model for increased precision in determining the effect of the test products on the scores. The adjusted means generated by this procedure compensate for any variations between treatment groups that existed in the baseline data. This reduces variability, increases power, and adjusts for imbalances at baseline due to subject attrition. Longitudinal (i.e., within-treatment) comparisons were performed for V-MI means using a one-sample t-test on the changes from baseline to the final examination. All comparisons were tested at an overall 0.05 level of significance using 2-sided tests.

### Safety assessments

An oral cavity examination was conducted at all visits including the gingival (free and attached), hard and soft palate, oropharynx, buccal mucosa, tongue, floor of the mouth, labial mucosa, mucobuccal/mucolabial folds, lips, and perioral area. In addition, subjects were asked if they had an any adverse events (AEs) since the prior visit. AEs were classified as serious or non-serious and by severity, as anticipated or unanticipated, and possible relationship to the test product. All AEs (examiner and self-report) were evaluated on subsequent visits to determine if AEs had resolved.

## Results

Forty (40) subjects were randomized and thirty-seven (37) completed the study. One patient was unwilling to comply with study procedures and the investigator removed the patient from the study and two patients withdrew consent. For randomized subjects, the gender distribution was 22 females and 18 males. The mean age was 50.4 years with the minimum age being 23 years and the maximum being 74 years. Caucasians represented 33 of the 40 subjects (82.5%) and none of the patients self-identified as Hispanic or Latino. Calculus baseline scores were comparable for all treatments. Subjects reported similar daily oral hygiene routines (twice daily).

### Efficacy

**Fig. 1:**
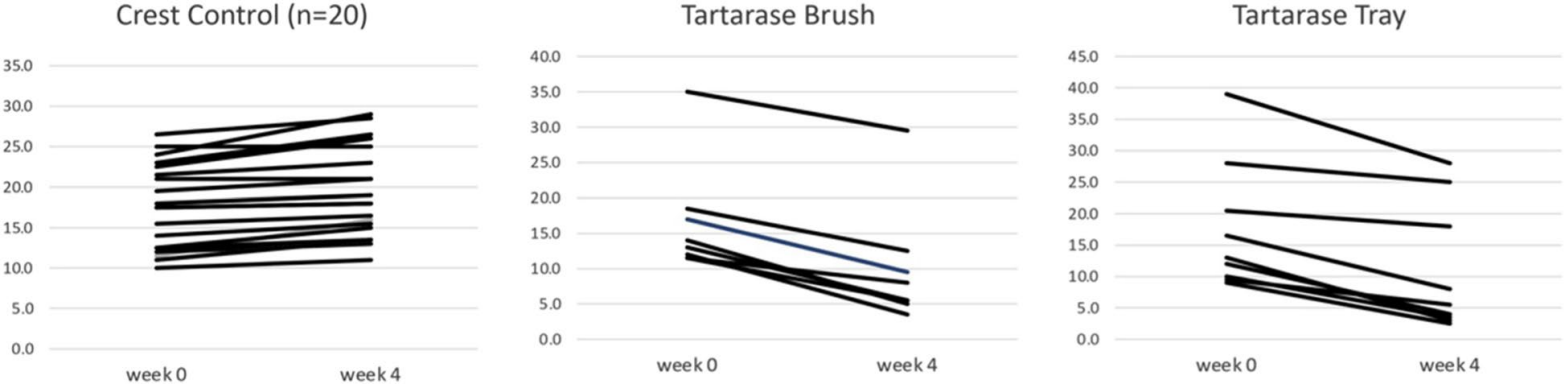
Individual changes in V-MI from baseline to week 4. Figure 1 displays the individual V-MI values at baseline and at 4 weeks for the different treatment groups

Both treatments with Tartarase reduced calculus over the 4-week study. Specifically, the Tartarase Brushing only group removed calculus by 40.0% and the Tartarase Brushing + Tray by 38.1%. The longitudinal intra group analysis indicated that the two Tartarase groups had statistically significant calculus reductions over the 4-week study (*p* < 0.001) and were statistically different from the Crest control group (*p* < 0.001), see Table 2. The results for both Tartarase treatment groups were comparable and all 17 assigned to the experimental product demonstrated a reduction in calculus (Fig. 1). Two subjects in the Tartarase brushing group failed to complete the study, one due to an adverse event that resolved in 5 days and one due to non-compliance. One subject in the Tartarase tray group withdrew due to a scheduling conflict.

**Table 2:** Change in V-MI from Baseline to Week 4

	Baseline V-MI Mean (SE)	Week 4 V-MI Mean (SE)	Change in V-MI Week 4 – Week 0 Mean (SE)	Change to Week 4 (%)
Tartarase Brushing	16.6 (2.79)	9.9 (2.98)	-6.7 (0.089)^a^	40.4% **reduction**
Tartarase Tray	17.5 (3.39)	10.8 (3.36)	-6.7 (0.082)^b^	38.1%** reduction**
Crest Control	17.3 (1.19)	19.4 (1.26)	2.1 (1.007)	12.2%** increase**

Table 2 lists the baseline, 4-week and change to V-MI mean and standard error (SE) and percent change and the direction of change in the V-MI of the different treatment groups

^a^ANCOVA result for Tartarase Brushing vs Crest Control *p* < 0.001

^b^ANCOVA result for Tartarase Tray vs Crest Control *p* < 0.001

In comparison, the Crest control group resulted in a statistically significant 12.2% increase in calculus over the 4-week study (*p* < 0.001). Moreover, the test subjects in the Crest control group had an increase in calculus deposition (Fig. 1). None of the test groups had a significant change at the 2-week examination evaluation.

### Oral cleanliness questionnaire

The Tartarase treatment group participants reported a more favorable response to how clean their teeth felt compared to the control group. The positive endorsements of ‘4’ or ‘5’ (5 = cleanest) were self-reported by 75% of the Tartarase Brush only and 67% of the Tartarase Brush + Tray group. The Control group reported a 50% positive endorsement to the two categories of ‘4’ or ‘5’. The test subjects were provided an opportunity to offer their overall impressions of the Tartarase product they were assigned on oral cleanliness including:

- *“I can feel each tooth in my mouth and they feel smooth”*
- *“I can feel my individual teeth better, my teeth—inside bottom teeth feel cleaner”*
- *“I feel like my mouth stays clean and fresh throughout the day. My breath smells better.”*

### Safety

No safety issues were noted from the use of Tartarase and there were no AEs nor any serious AEs. Tartarase was well tolerated. There were 5 AEs based upon oral examination (4 AEs were attributed to toothbrush abrasion and the other was an inflamed papilla (mild) on anterior of the tongue). In total, the control group had 1 AE, there were 3 AEs in the Tartarase brushing group and 1 AE in the Tartarase Tray group. There were no oral hard tissue changes noted during the 4-week trial. Three test subjects self-reported AEs with 2 who listed gum or lip sensitivity (Tartarase tray group) and 1 experienced a burning tongue (Tartarase Brushing group). All AEs (Examiner observation and self-reported) resolved within the 5-day re-evaluation period.

## Discussion

Tartarase significantly reduced dental calculus after 4 weeks with approximately a 40% reduction in both Tartarase test groups while the Crest Control group demonstrated a 12% increase of dental calculus deposits. According to our literature review, this is the first reported toothpaste to significantly remove dental calculus at 4 weeks. Moreover, every Tartarase test subject had a decrease in dental calculus after 4-weeks of daily, unsupervised use. This pilot proof of principle study provides support to our proposed mechanism of action that DNA and proteins provide integral and strategic structural support of dental calculus, allowing enzymes that target DNA and denatured proteins to be effective in disrupting the structure of dental calculus and reducing dental calculus accumulation. Equally important is the evidence of no further calculus build up, suggesting that the Tartarase formula may be effective in preventing calculus formation following a professional dental cleaning. The fact that the Tartarase test subjects demonstrated an approximate 40% calculus reduction in 4 weeks while the control group experienced a 12% increase in calculus suggests that a dose response study is recommended in a future study, already in the planning stages. A lower enzyme dose may prove efficacious at both prevention of calculus accumulation and removal of pre-existing calculus. There are several limitations to the current 4-week proof-of-principle study. First, it is not known how fast all dental calculus would be eliminated once the structural integrity is disrupted. Moreover, a long-term study (6-months) using test subjects who have not had a dental cleaning in the past year would determine the efficacy and safety used daily for the normal period between cleanings. As the active ingredients in Tartarase are enzymes, they need to be in contact with tartar to have their desired effect and the correct dosage needs to be determined as well as the proper delivery vehicle. This was the justification to fabricate a custom tray as an applicator. To elaborate on this principle, conventional toothpastes, that have abrasives as their dominant active ingredient in combating calculus accumulation, act only at the site where the brushing process is taking place. Enzymes, on the other hand, once deposited on the calculus, continue to work to weaken and dismantle calculus until they become inactive through processes like product inhibition or protein denaturation. Additionally, the enzymes afford other advantages, such as being accessible to the spaces between the teeth that are inaccessible to abrasives and toothbrush bristles, and their size (3000 × smaller than conventional abrasives) will allow them access to the interstices within the calculus itself.

Abrasives have been the most common toothpaste ingredients for addressing the gritty feel that the tongue experiences in touching the developing calculus on the tooth surfaces. In the investigation [11] in which the study subjects were initially examined for 3 months to assess the effectiveness of their oral hygiene habits, followed by scaling, and 6 months later assessed for several parameters, calculus accumulation being most relevant to the Tartarase study being presented here. Figure 4 in [11] demonstrates that 6 months following scaling and use of the Dr. D-Tart toothpaste, calculus accumulation, assessed by the Volpe-Manhold calculus index, increased by 0.87 mm. This was determined by using ruler to measure the bar height, relative to the index scale. This was surprising, considering the results of the 3-month assessment period, preceding the scaling, in which the Dr. D-Tart study group experienced a 0.51 mm decrease in calculus. The fact that the Dr. D-Tart study groups increased calculus following scaling but had a decrease in calculus prior to scaling is difficult to square with the conventional models of calculus formation, where plaque initiates calculus formation, and would appear to be more fragile, and thus more susceptible to abrasion. The authors do not discuss this.

The oral cleanliness questionnaire did not provide statistically significant differences between the control and test products but there were numerical preferences for Tartarase in mouth feel and breath improvements. The opportunity to gather self-reported data comparisons with Crest provided assurance that enzymes can be added to a dentifrice without creating a product that is offensive to the consumer. This proof of principle pilot study used twice the level of Tartarase tested in vitro and the questionnaire data provided a positive experience (non-negative) with this enzyme system.

There is an additional aspect of this type of oral hygiene formulation that needs to be addressed. As the active ingredients of Tartarase are enzymes, a general feature of enzymes is important to consider. Enzymes are protein catalysts. They facilitate a chemical reaction that would normally occur to happen at an enhanced velocity. In addition, they allow the reaction to occur in both forward and reverse directions. Thus, with these types of enzymes the accumulating products inhibit the enzymes from catalyzing their forward reactions. We speculate this to be the reason why the Tartarase tray group did not out-perform the Tartarase brushing group. In addition, our in-vitro studies have found that the enzymes act extremely quickly, with the reactions completed in < 5 min, indicating that the 30-min incubation time in the tray group provided no advantage.

In our pre-clinical studies, we have data to support the importance of a two-enzyme formulation. It is rational that the only way calculus can be attacked is at its outer 2-dimensional surface. Our model of calculus structure is a calcified complex of fundamental biopolymers entrapping bacteria and random debris. If an enzyme that has specificity for only one of the complex biopolymers, then, when the surface accessible target biopolymers has been consumed, the enzyme can go no further as the remaining target is concealed by biopolymers that are not targets for the single enzyme. Thus, using the enzymes in combination allows for a progressive mechanism that systematically peels away calculus from its outermost surface. With continued application it is conceivable that the process could eventually reach the tooth enamel.

Our clinical study has demonstrated a capacity to reduce calculus and further clinical studies may be designed to investigate the relationship between calculus accumulation and periodontal disease. If this association can be established, further investigations to elucidate mechanistic relationships between systemic diseases and periodontal disease can be undertaken.

Lastly, the single AE noted (erythema) by the examiner was most likely caused by brushing 4X per day in the Tartarase brushing group (vs. 2X in the Crest control group). The exaggerated brushing frequency will not be recommended in future human clinical trials.

## Conclusion

The prototype Tartarase toothpaste demonstrated a significant calculus removal in a 4-week trial period with test subjects who were known calculus-formers that had their teeth cleaned between 2 and 6 months prior to entering the research study. This proof-of-principle trial suggests that Tartarase has the potential to reduce calculus when used in a daily routine of oral hygiene as safe over-the-counter formulation.

Our study supports the model that calculus is partially composed of biopolymers that become mineralized, in addition to interdigitated denatured and occasionally crosslinked proteins, that are integral to calculus structure and are surface accessible to enzymatic digestion. Thus, if the enzymes can remove sufficient calculus that cannot be replaced in a roughly 12-h time span between brushings with Tartarase, there will be a progressive loss of calculus.

## Data Availability

The datasets used and/or analyzed during the current study are available from the corresponding author on reasonable request.

## Abbreviation

V-MI: Volpe-Manhold Index
